# Physical functioning factors predicting a return home after stroke rehabilitation: A systematic review and meta-analysis

**DOI:** 10.1177/02692155231185446

**Published:** 2023-07-10

**Authors:** Odile Chevalley, Steven Truijen, Emmanuelle Opsommer, Wim Saeys

**Affiliations:** 1 School of Health Sciences (HESAV), University of Applied Sciences and Arts Western Switzerland (HES-SO), Lausanne, Switzerland; 2 Department of Rehabilitation Sciences and Physiotherapy, Faculty of Medicine and Health Sciences, 81844 University of Antwerp, Antwerp, Belgium

**Keywords:** Functional status, motor activity, patient discharge, rehabilitation, stroke

## Abstract

**Objectives:**

This systematic review and meta-analysis sought to identify the physical functioning factors associated with home discharge after inpatient stroke rehabilitation.

**Data sources:**

A search of PubMed, Embase, CINHAL, The Cochrane Library (Trials), Web of Science, and PEDro were conducted up until May 2023.

**Methods:**

Two independent reviewers selected studies for population (patients with stroke), predictive factors (physical functioning), outcome (discharge destination), setting (inpatient rehabilitation), and study designs (observational and experimental studies). Predictive factors were identified among assessments of the “body function” and “activity” components of the International Classification of Functioning. Methodological quality was assessed with the Newcastle-Ottawa Scale. The findings used quantitative and narrative syntheses. Meta-analyses were performed with the inverse variance method and the random-effects model using included studies with sufficient data.

**Results:**

Forty-five studies were included with 204,787 participants. Included studies assessed the association of independence in activities of daily living, walking, rolling, transferring, and balance on admission with a probability of returning home. Motor (odds ratio = 1.23, 95% confidence interval: 1.12–1.35, *p* < .001) and total (odds ratio = 1.34, 95% confidence interval: 1.14–1.57, *p* < .001) Functional Independence Measure scores on admission were significantly associated with home discharge in meta-analyses. Additionally, included studies showed that independence in motor activities, such as sitting, transferring, and walking, and scores above thresholds for the Functional Independence Measure and Berg Balance Scale on admission were associated with discharge destination.

**Conclusion:**

This review showed that higher independence in activities of daily living on admission is associated with home discharge after inpatient stroke rehabilitation.

## Introduction

After acute care, half of the patients with stroke (53.2%) are admitted to inpatient rehabilitation for further recovery.^1^ About 80% of these patients can return home upon discharge, but 20% must be admitted to long-term care facilities.^2^ During inpatient rehabilitation, health professionals rely on clinical examination, expertise, and predictive factors to identify appropriate rehabilitation goals with the patients. Predictive factors have been identified for motor, sensation, cognition, and language recovery.^3^ Health professionals need to know which factors are commonly associated with the ability to return home after stroke to guide discharge planning decisions, facilitate patient flow between settings, and enhance bed availability in rehabilitation units.

Previous systematic reviews focused on predictive factors after acute care^4,5^ or for the context of the United States of America.^6^ They identified that discharge destination is predicted by age, poststroke functionality, admission to a university hospital, cognitive independence, prestroke household situation, marital status, insurance, and geographical situation after acute care^4,5^; and that good poststroke functionality, assessed with the Functional Independence Measure, and lower stroke severity are predictive for a home discharge in the United States of America.^6^ A recent systematic review explored socio-environmental factors after inpatient stroke rehabilitation and identified the high probability of a return home for patients living at home and with relatives prestroke, benefiting from support at home, and being married.^7^ Conversely, another systematic review showed older age and greater stroke severity to be predictive of a long-term care facility discharge.^2^ In addition to these factors, it stated that potentially modifiable factors should be evaluated regarding discharge destination. Physical functioning factors that can be improved by physical rehabilitation are included in these modifiable factors.

We focused on physical functioning factors that may predict a discharge home after inpatient rehabilitation. Functional Independence Measure assessing the motor and cognitive function of activities of daily living might be one of these, but individual physical functions also need to be investigated. Over the last decades, various physical functions have been evaluated, such as balance^8^ and rolling or walking ability,^9^ but no review systematically reported the effects of these factors on discharge destination. Therefore, this systematic review aimed to identify physical functioning factors associated with the discharge home of patients with stroke after inpatient rehabilitation.

## Methods

This review was reported following the Preferred Reporting of Items in Systematic Reviews and Meta-Analysis (PRISMA) guidelines^10^ (Supplemental File 1). This systematic review was registered in the PROSPERO database (CRD42021158690).

Observational and experimental studies were eligible if they included adults (>18 years) with stroke who were admitted to inpatient stroke rehabilitation after acute care and reported on at least one physical functioning outcome measure and discharge destination. Inpatient rehabilitation must have represented a temporary setting for stroke rehabilitation between acute care and the return home, typically during the subacute poststroke phase.^11^ Studies must have reported on physical functioning assessed on admission or on discharge to be included. Physical functioning includes any motor function of the lower or upper limbs, trunk function, and function during complex activities (i.e. activities of daily living).^12^ Activities of daily living assessments were included when the majority of the scale assessed motor function. Physical functioning assessments include but are not limited to, the 10-meter walking test, Berg Balance Scale, Functional Independence Measure, and Barthel Index. Studies were eligible when they assessed the discharge destination at the end of rehabilitation and when the home was a discharge destination. The home was defined as an independent living situation where no constant presence of a professional caregiver was mentioned.^7^ Observational and experimental study designs were eligible. Finally, studies published in peer-reviewed journals in English, Dutch, German, or French were eligible.

Studies reporting no physical functioning outcomes were excluded. In addition, studies were excluded when participants were discharged from an acute care setting when participants were not discharged home, and when no data about the probability of home discharge were reported. Systematic reviews and meta-analyses were excluded.

We systematically searched studies published until May 2023 in six databases, including PubMed, Embase, CINHAL, The Cochrane Library (Trials), Web of Science, and PEDro. The search string was adapted to each database and included keywords for stroke, discharge planning, and physical functioning (Supplemental File 2). Additionally, we screened the reference lists of included studies for additional studies.

Two reviewers independently screened the studies for inclusion in the systematic review in two steps: first, on title and abstract; second, on full text. Any disagreement between reviewers about study inclusion was discussed until a consensus was reached for each step of the selection process. After including the studies in the review, a data-extraction table was used to collate data concerning participants, methods, outcomes, and predictive values. Every assessment of physical functioning was extracted with their results to predict discharge destination either on admission or discharge in a table.

Two reviewers independently assessed the methodological quality of included studies with the Newcastle-Ottawa Scale for cohort and case-control studies.^13^ This scale assesses the selection of the study group, comparability of the groups, and ascertainment of exposure for cohort studies, and the selection of the study group, comparability, and outcome of interest for case-control studies. The face validity of the Newcastle-Ottawa Scale is established,^13^ and the interrater reliability is fair for the overall score.^14^

We reported mean and standard deviation or median and interquartile range as appropriate for descriptive data. We pooled results for predictive factors when at least three studies reported results for the same outcome measure at the same assessment time (admission or discharge). We used odds ratio and confidence interval, logistic regression coefficient and standard error, or the number of events for each group for the analyses. The odds ratio and adjusted odds ratio were analyzed separately.^15^ We pooled adjusted odds ratio together when the studies adjusted at least for one of the following factors: age, gender, prestroke living situation, family support, and length of stay. We used the inverse variance method with a random-effects model for the meta-analyses to calculate pooled odds ratios and 95% confidence intervals. The random-effects model was preferred because clinical heterogeneity was present among included studies. We performed sensitivity analyses by removing outliers from meta-analyses. Analyses were performed with Cochrane's Review Manager.^16^ When a meta-analysis was not possible, the outcomes were reported narratively.

We grouped the various assessments of physical functioning together according to the components of the International Classification of Functioning. We divided the assessments focused on the activity component into assessments of activities of daily living and assessments of motor activities. Assessments of activities of daily living included the Functional Independence Measure, Barthel Index, Katz-15, Kenny Self-Care Scale, Lucerne ICF-based Multidisciplinary Observation Scale, and Frenchay Activities Index. Assessments of motor activities included the Trunk Control Test, a revised version of the Ability for Basic Movement Scale, Motor Assessment Scale, Berg Balance Scale, and assessments of balance, walking, rolling, and transferring. We reported assessments focused on both body function and structure as well as activity components, including the Chedoke McMaster Stroke Assessment and Simplified Stroke Rehabilitation Assessment of Movement. Finally, we reported the assessments focused on body function and structure components, including the Motricity Index, Fugl-Meyer Assessment, Stroke Impairment Assessment Set-motor function, and hand-grip strength.

## Results

**Figure 1.**
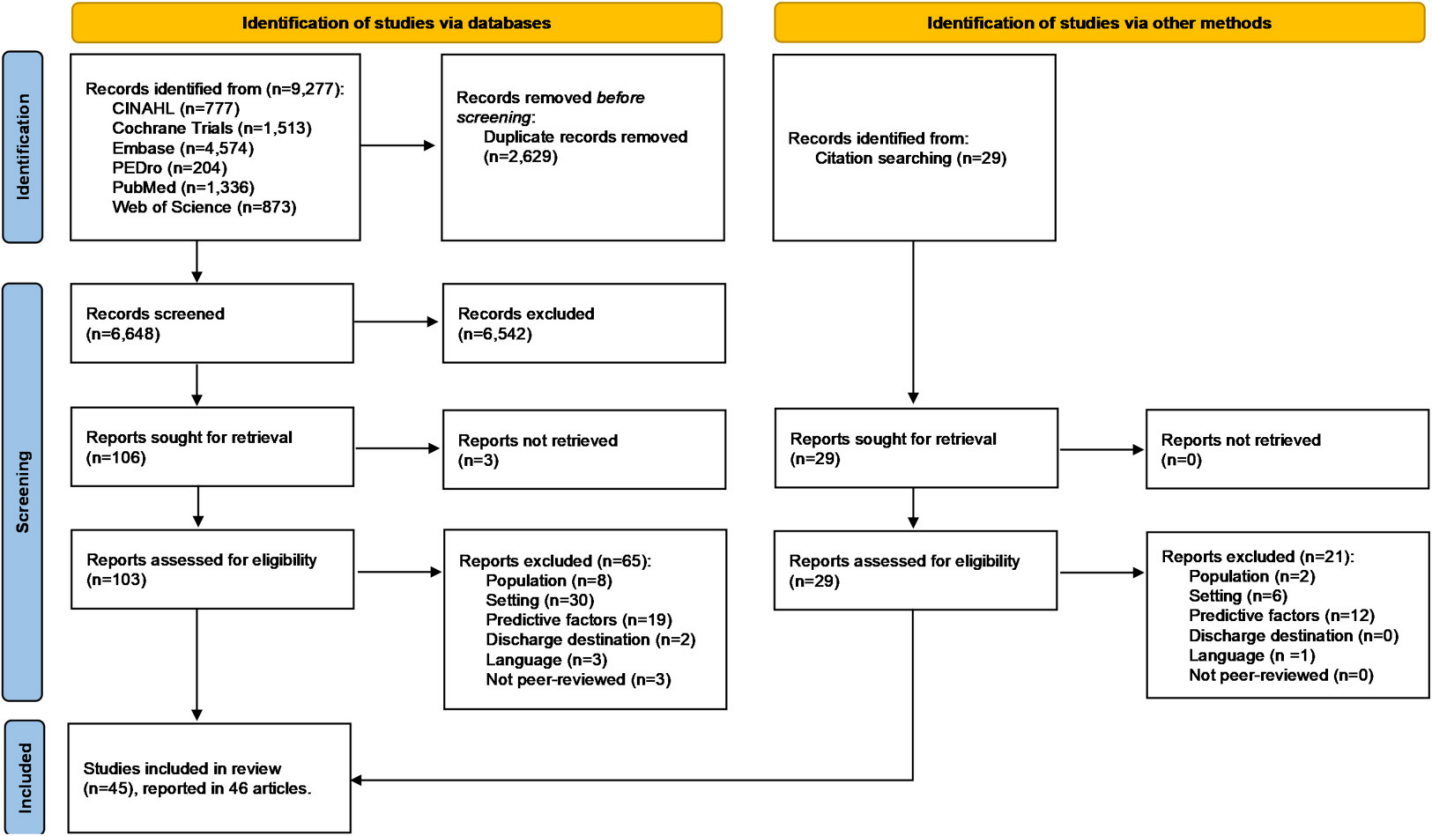
Flow diagram for the selection process.

From the 6648 non-duplicate records identified, 38 studies were selected. Two studies^17,18^ reported on the same patient population and were counted as one. This meant 37 studies were initially selected. After a reference list manual search, eight additional studies were included. Thus, 45 studies were included in the systematic review (Figure 1).

The characteristics of the included studies are reported in Table 1. There were 43 cohort studies and two case-control studies. The studies were conducted in North America (*n* = 22), Europe (*n* = 9), Asia (*n* = 12), and Australia (*n* = 2). In total, 204,787 patients were represented in the studies included in this review. Clinical heterogeneity was observed in sample size, mean age of participants, length of stay in the rehabilitation setting, and length of stay in the acute care setting. Additionally, various physical function outcome measures predicting discharge destination were reported (see outcome measures in Table 1). In the included studies, 144,624 patients (71.2%) were discharged home, and 58,339 patients (28.7%) were discharged to other destinations.

**Table 1.** Characteristics of included studies.

Study ID by country	Sample size, *n* (% men)	Age, mean ± SD	Stroke type, *n* (%)		Functional Independence Measure score on admission, mean ± SD or *median* (*IQR*)	Onset to admission interval, mean ± SD in days or *median* (*IQR*)	Length of stay, mean ± SD in days or *median* (*IQR*)	Discharge destination, *n* (%)	Outcome measures
			Ischemic	Hemorrhagic					
**Australia**									
Brauer 2008	566 (54.1)	72.5 ± 13	*Not reported*		*Not reported*	15.3 ± 16	40.1 ± 35	Home: 410 (72.4) Other: 136 (24.0) Death: 8 (1.4) Unknown: 12 (2.1)	Motor Assessment Scale (items 1-Rolling and 5-Gait) *On admission*
Tucak 2010	239 (52.3)	78.1 ± 7.1	193 (80.8)	46 (19.2)	*Not reported*	17.9 ± 12.4	32.6 ± 20.7	Home: 169 (70.7) Other: 70 (29.3)	Motor Assessment Scale *On admission*
**Canada**									
Agarwal 2003	104 (48.1)	72.0 ± 10.1	94 (90.4)	10 (9.6)	Home: 87.0 ± 19.4 Other: 68.8 ± 19.3	Home: 18.6 ± 18.3 Other: 21.1 ± 10.9	Home: 31.1 ± 17.9 Other: 50.7 ± 26.9	Home: 80 (76.9) Other: 24 (23.1)	Admission Ambulatory status, Chedoke McMaster Stroke Assessment, Functional Independence Measure (total, items), Berg Balance Scale
Oczkowski 1993	113 (52.2)	65.8	100 (92.6)	8 (7.4)	*Median 80*	*Median 52*	*Median 64*	Home: 80 (70.8) Other: 30 (26.5) Death: 3 (2.7)	Functional Independence Measure, Chedoke McMaster Stroke Assessment
Saab 2019	227	*Not reported*	227 (100)		*Not reported*	*Not reported*	*Not reported*	*Not reported*	Berg Balance Scale and Functional Independence measure *On admission and discharge*
Tanwir 2014	268 (51)	*Not reported*	*Not reported*		*Not reported*	*Not reported*	*Not reported*	*Not reported*	Motor Functional Independence measure *On admission*
Teasell 2005	196 (53.1)	72 ± 11	166 (84.7)	30 (15.3)	*46* (*IQR 20*)	56 ± 33	88 ± 39	Home: 85 (43.4) Other: 104 (56.6)	Functional Independence Measure *On admission*
Wasserman 2020	240 (60.4)	Home: 64 ± 13 Other: 75.5 ± 11.5	205 (85.4)	32 (13.3)	Home: 79.8 ± 22 Other: 52.9 ± 16.1	Home: 23.9 ± 18.1 Other: 26.1 ± 16.7	Home: 53.9 ± 25.3 Other: 112.4 ± 61.7	Home: 180 (75) Other: 60 (25)	Functional Independence Measure (total, subscores), Berg Balance Scale (measurability, total) *On admission*
Wee 1999	128 (61.7)	69.9 ± 11.6	*Not reported*		Home: 88.5 ± 25 Other: 57.1 ± 27	28.7 ± 26.5	(IQR-29–66)	Home: 98 (76.6) Other: 30 (23.4)	Berg Balance Scale *On admission*
Wee 2003, 2005^a^	313 (51.8)	76 ± 8	278 (88.8)	35 (11.2)	87.8	37 ± 22	*Not reported*	Home: 246 (78.6) Other: 67 (21.4)	Berg Balance Scale for subjects without family support, Berg Balance Scale *On admission*
**China**									
Ling 2004	1111 (56.9)	70.4 ± 11.6	*Not reported*		69.2 ± 24.1	*Not reported*	36.6 ± 20.2	Home: 789 (71) Other: 322 (29)	Functional Independence Measure (subscores) *On admission*
**France**									
Petrilli 2002	93 (49.5)	64.8	72 (77.4)	21 (22.6)	H: 85.37 ± 27.6 O: 54.7 ± 31.57	17.9 ± 12.5	*Not reported*	Home: 81 (87.1) Other: 11 (11.8) Death: 1 (1.1)	Functional Independence Measure
**Hong Kong**									
Li 2020	562 (52.3)	Home: 69.8 ± 13.6 Other: 74.1 ± 12.9	373 (66.4)	136 (24.2)	Home: 68.8 ± 25.8 Other: 50.0 ± 26.7	Home: 10.7 ± 9.4 Other: 11.1 ± 10.5	Home: 31.9 ± 20.5 Other: 39.7 ± 25.4	Home: 304 (54.1) Other: 258 (45.9)	Total and motor Functional Independence Measure *On admission*
			Unspecified: 53 (9.4)						
**Israel**									
Davidoff 1992	192 (59.9)	Home: 60.9 Other: 65 ± 7	*Not reported*		Home: 43.6 ± 15.7 Other: 13.1 ± 4.2	Home: 27.8 ± 16.6 Other: 24.3 ± 15.9	Home: 106.7 ± 64.2 Other: 168.7 ± 97.1	Home: 141 (73.4) Other: 51 (26.6)	Kenny self-care
**Italy**									
Denti 2008	359 (37.9)	80.8 ± 4.7	286 (79.7)	64 (17.8)	55.8 ± 24	22.3 ± 14.6	50.0 ± 27.7	Home: 287 (79.9) Other: 72 (20.1)	Total and motor Functional Independence Measure *On admission*
			Unspecified: 9 (2.5)						
Gialanella 2012	241 (48.5)	71.1 ± 10	195 (80.9)	46 (19.1)	54 ± 22	17.6 ± 5.9	49.4 ± 16	Home: 205 (85.1) Other: 36 (14.9)	Functional Independence Measure (subscores), Fugl-Meyer, Trunk Control Test
Massucci 2006	997 (51.5)	69.8 ± 12	753 (75.8)	227 (22.8)	*Not reported*	26.1 ± 25.5	52.4 ± 32.9	Home: *not reported* Other: *not reported* Death: 34 (3.4)	Trunk Control Test, Motricity Index
**Japan**									
Hirano 2017	80 (62.5)	62.7 ± 11.6	19 (23.8)	55 (68.8)	15.5 ± 14.9 (BI)	30.6 ± 15.2	106.6 ± 44.5	Home: 61 (76.3) Other: 19 (23.7)	Trunk Control Test, the ratio of knee extensor strength on the non-paralyzed side to body weight
			Unspecified: 6 (7.5)						
Ito 2022	1229 (59.2)	68.7 ± 13.5	735 (59.8)	494 (40.2)	*Median 78* (*54–96*)	32.2 ± 12.7	88.5 ± 45.3	Home: 1011 (82.3) Other:218 (17.7)	Motor Functional Independence Measure, grip strength, Stroke Impairment Assessment Set-motor function *On admission*
Koyama 2011	163 (60.7)	69.71 ± 12	101 (62.0)	62 (38.0)	52.55 ± 22.74	34.3 ± 14.3	101.6 ± 40.1	Home: 123 (75.5) Other: 40 (24.5)	Motor Functional Independence Measure *On admission and discharge*
Maeshima 2016	89 (65.2)	61.9 ± 11.9	*Not reported*		*Home: median 64* *Other: median 30.5*	30.8 ± 17.2	70.7 ± 31.8	Home: 71 (79.8) Other: 18 (20.2)	Total and motor Functional Independence Measure *On admission and discharge*
Matsushita 2022	699 (52.6)	*Median 79*	484 (69.2)	215 (30.8)	*Men: median 69* (*38–91*) *Women. Median 63* (*33–86.5*)	*Men: median 24* (*19–31*) *women: median 22* (*17–31*)	*Men: median 88.5* (*53–139.25*) *women: median 92* (*61–130.5*)	Home: 448 (64.1) Other: 251 (35.9)	Grip strength
Miura 2018	342 (58.8)	69 ± 13	203 (40.6)	139 (59.4)	*Not reported*	138.3 ± 53.5	*Not reported*	Home: 175 (51.2) Other: 167 (48.8)	Berg Balance Scale, total and motor Functional Independence Measure
Mutai 2012	174 (51.1)	73.0 ± 10.8	106 (60.9)	68 (39.1)	72.6 ± 27.6	33.5 ± 18.6	Home: 58.4 ± 40.8 Other: 50.5 ± 34.4	Home: 151 (87) Other: 23 (13)	Motor Functional Independence Measure *On admission*
Onishi 2022	205 (60.0)	76.8 ± 8.8	134 (65.4)	71 (34.6)	58.5 ± 29.8	40.1 ± 18.7	70.9 ± 35.4	Home: 147 (71.7) Other: 58 (28.3)	Motor Functional Independence Measure *On discharge*
Yang 2020	94 (43.6)	Home: 75.5 ± 11.9 Other: 78.4 ± 9.5	69 (73.4)	25 (26.6)	Home: 76.3 ± 25.18 Other: 41.55 ± 15.41	Home: 26.5 ± 12.1 Other: 30.4 ± 13.3	Home: 55.4 ± 29.8 Other: 89.4 ± 44.1	Home: 61 (64.9) Other: 33 (35.1)	Revised version of the Ability for Basic Movement Scale
**The Netherlands**									
Vluggen 2020	92 (48.9)	79 ± 6.4	*Not reported*		*Not reported*	*Not reported*	*Not reported*	Home: 71 (77.2) Other: 21 (22.8)	Katz-15, Frenchay Activities Index
**Sweden**									
Löfgren 1997	100 (49)	75.7 ± 8.1	76 (76)	24 (24)	*Not reported*	*18* (*IQR 14–28*)	*58* (*IQR 31–90*)	Home: 93 (93) Other: 7 (7)	Fugl-Meyer
Löfgren 2000	116 (47)	75.2 ± 8.5	94 (81)	22 (19)	*Not reported*	*17* (*IQR 13–23*)	*36* (*IQR 21–59*)	Home: 71 (61) Other: 31 (27) Death: 14 (12)	Katz Activity of Daily Living Index, Postural stability score, Motricity Index
**Switzerland**									
Frank 2010	1332 (49.3)	*Median 76.5*	*Not reported*		Home: 82.7 ± 24.1 Other: 49.7 ± 25.8^b^	19.8 ± 13.6	50.9 ± 37	Home: 828 (62.2) Other: 469 (35.3) Death: 35 (2.6)	Sitting and standing balance, walking ability, Functional Independence Measure (subscores) *On admission*
Ottiger 2020	555 (58.4)	*68* (*IQR 57–77*)	395 (71.2)	160 (28.8)	*Not reported*	*9 (IQR 7–15)*	*Not reported*	Home: 468 (84.3) Other: 87 (15.7)	Lucerne ICF-based Multidisciplinary Observation Scale *On discharge*
**United States of America**									
Alexander 1994	464	72,2 ± 12,8	*Not reported*		*Not reported*	18.3	33.8	Home: 361 (77.8) Other: 103 (22.2)	Admission Functional Independence Measure
Black 1999	234 (56)	68.8 ± 13	*Not reported*		69.7 ± 19.4	*Not reported*	19.3 ± 10.4	Home: 168 (72) Other: 66 (28)	Functional Independence Measure
Bottemiller 2006	748 (57)	69 ± 13	499 (66.7)	249 (33.3)	*Not reported*	*Not reported*	*Not reported*	Home: 489 (65.4) Other: 259 (34.6)	Functional Independence Measure *On admission and discharge*
Brown 2015	148,367 (48)	70.6 ± 13.1	143,916 (97)		56.8 ± 19.5	8.2 ± 12	16.5 ± 9.9	Home: 103,857 (70) Other: 44,510 (30)	Motor Functional Independence Measure *On admission*
Granger 1992	7905	70.7	*Not reported*		*Not reported*	22.2 ± 32.2	32 ± 22.4	Home: 5909 (74.8) Other: 1769 (22.4) Death: 79 (0.1)	Functional Independence Measure *On discharge*
Mokler 2000	259 (53.3)	64	*Not reported*		*Not reported*	18.5	25.9	Home: 115 (44) Other: 144 (56)	Functional Independence Measure (subscores)
Ng 2005	89 (54)	71.5 ± 13.1	81 (91.0)	8 (9.0)	65.0 ± 25.3	11.4 ± 8.8	29.2 ± 20.9	Home: 55 (62) Other: 32 (36) Death: 2 (2)	Functional Independence Measure *On discharge*
Nguyen 2015	2085 (50.6)	Home: 63.7 ± 13.9 Other: 69.9 ± 12.5	1750 (83.9)	335 (16.1)		Home: 9.8 ± 10.5 Other: 11.9 ± 12.4		Home: 1631 (78.2) Other: 454 (21.8)	Motor Functional Independence Measure
Ouellette 2015	407	68.2 ± 13.9	309 (76)	98 (24)	Home: 50.8 ± 15.6 Other: 39.1 ± 13.4^b^	19	20 ± 9.5	Home: 297 (73) Other: 110 (27)	Functional Independence Measure (total, subscores) and Simplified STroke REhabilitation Assessment of Movement *On admission*
Pereira 2014	189 (54.5)	68.8 ± 14.4	*Not reported*		50.4 ± 11.4	*Not reported*	63.4 ± 30.7	Home: 124 (65.6) Other: 65 (34.4)	Functional Independence Measure *On admission*
Pohl 2013	31,919 (43)	77.1 ± 7.3	*Not reported*		60 ± 19.6	*Not reported*	*Not reported*	Home: 24,035 (75.3) Other: 7884 (24.7)	Functional Independence Measure *On admission and discharge*
Ween 1996	376 (45)	73 ± 12	320 (85)	34 (9)	Home: 69.8 Other: 48.4	16 ± 30	33 ± 22	*not reported*	Functional Independence Measure *On admission*
			Unspecified: 22 (6)						
Ween 2000	244	73 ± 11	114 (46)	22 (9)	69 ± 20	9 ± 6	30 ± 15	Home: 167 (68.4) Other: 77 (31.6)	Admission Functional Independence Measure, functional status at discharge
			Unspecified: 108 (45)						
Wilson 1991	282 (47.9)	69	*Not reported*		H: 56.4 O: 43.6	22	H: 32.8 O: 23.1^c^	Home: 212 (75.2) Other: 70 (24.8)	Functional Independence Measure *On admission and discharge*

IQR: interquartile range; *N*: number of participants; SD: standard deviation.

^a^ The studies reported on the same study sample.

^b^ Mean FIM score on admission for the entire sample: Frank 2010 (70.2 ± 29.5) and Ouellette 2015 (47.6 ± 15.9).

^c^ Mean LOS for the entire sample: Wilson 1991 (31).

The methodological quality of the two case-control studies^19,20^ was moderate (scores 5 and 6 out of 9), showing the risk of bias for comparability of cases and controls and the ascertainment of exposure in both studies and for the adequacy of case definition in one study.^19^ The methodological quality of cohort studies^8,9,17,18,21–59^ was moderate to high (score from 6 to 9 out of 9, except for one study that score 4 out of 9),^60^ showing risk of bias for the comparability of the groups, primarily because no adjustment for confounders was reported, for the representativeness of the exposed cohorts, assessment of outcomes, adequacy of follow-up of cohorts, and ascertainment of exposure (Supplemental File 3).

**Figure 2.**
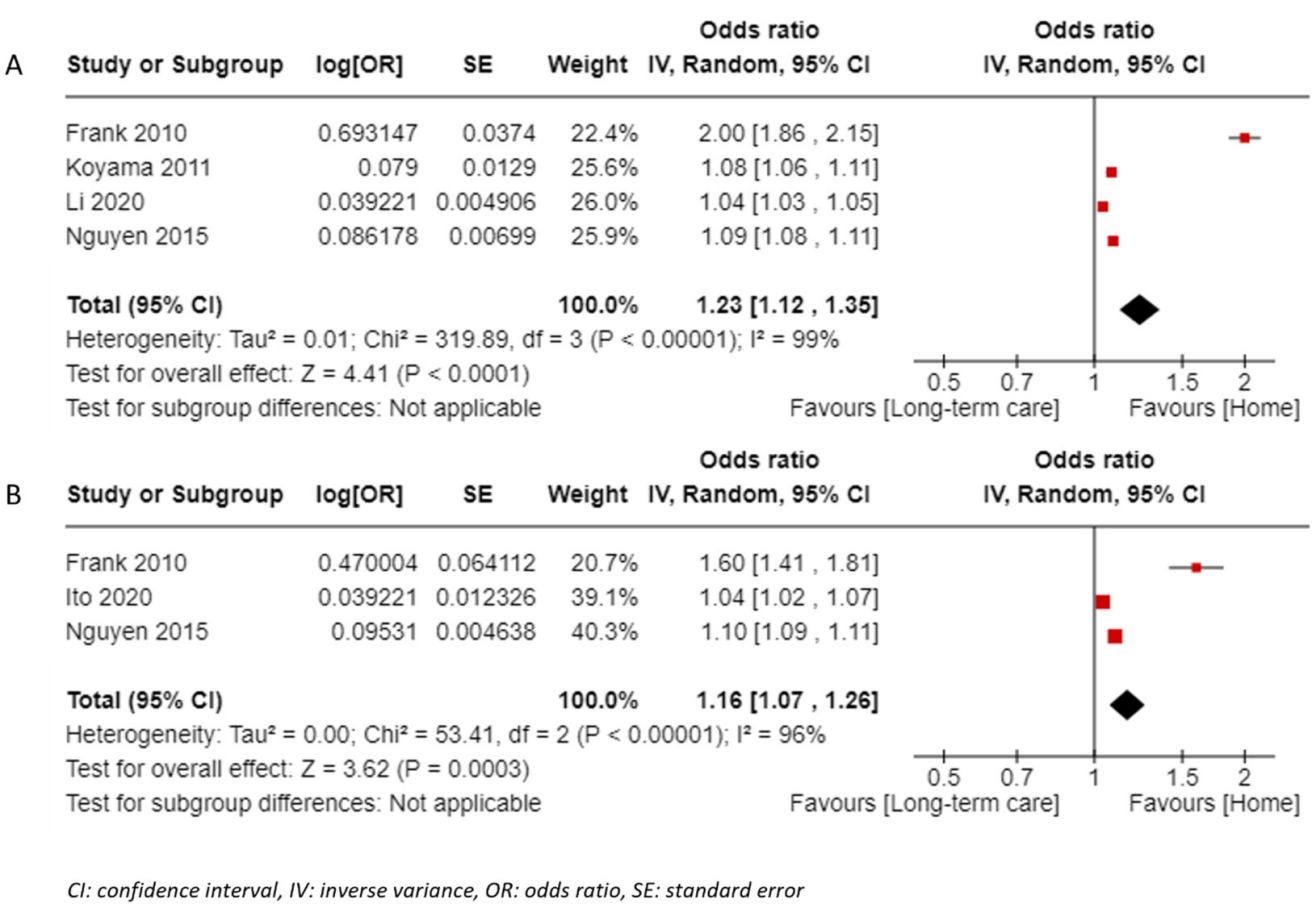
Forest plot for the motor Functional Independence Measure on admission to predict returning home: (a) pooled results of studies reporting odds ratio and (b) pooled results of studies reporting adjusted odds ratio.

*Motor activities of daily living on admission:* From 13 studies^20,25–28,31,32,38,39,41,44,49,57^ assessing the motor Functional Independence Measure, four studies^27,31,32,41^ of moderate and high quality (score 9 *n *= 2, score 8 *n *= 1, score 7 *n *= 1) were included in the meta-analysis of odds ratio (Figure 2(a)) and showed significant results with high heterogeneity (odds ratio = 1.23, 95% confidence interval: 1.12–1.35, *I*^2 ^= 99%). The result was also significant when the outlier^27^ was removed (odds ratio = 1.07, 95% confidence interval: 1.03–1.11, *I*^2 ^= 95%). Three studies^20,27,57^ of high quality (score 9 *n *= 2, score 8 *n *= 1) were included in the meta-analysis of adjusted odds ratio (Figure 2(b)) and showed significant pooled results (odds ratio = 1.16, 95% confidence interval: 1.07–1.26, *I*^2^ = 96%). Apart from meta-analyses (Supplemental File 4), five studies^20,25,28,36,39^ showed positive results of the motor Functional Independence Measure and one study^26^ showed no predictive value of the scale for home discharge. The threshold values (≥ 26)^44^ showed a significant probability of discharge home for patients with a score about it. Subgroup comparison showed that patients with intermediate (39–50 or 27–52) or high scores (≥ 51, ≥ 53) were significantly more likely to return home than patients with low scores.^32,49^ Self-care and locomotion motor subdomains of Functional Independence Measure were significant for a home discharge in univariate analysis, but contradictory results were present in multivariate analyses.^27,33^ Additionally, two other scales (Katz-15 and Barthel Index mobility subscore) showed significant results^35,52,61^ with the discharge destination, whereas one scale (Kenny Self-Care Scale) showed nonsignificant results regarding the discharge destination.^19^

**Figure 3.**
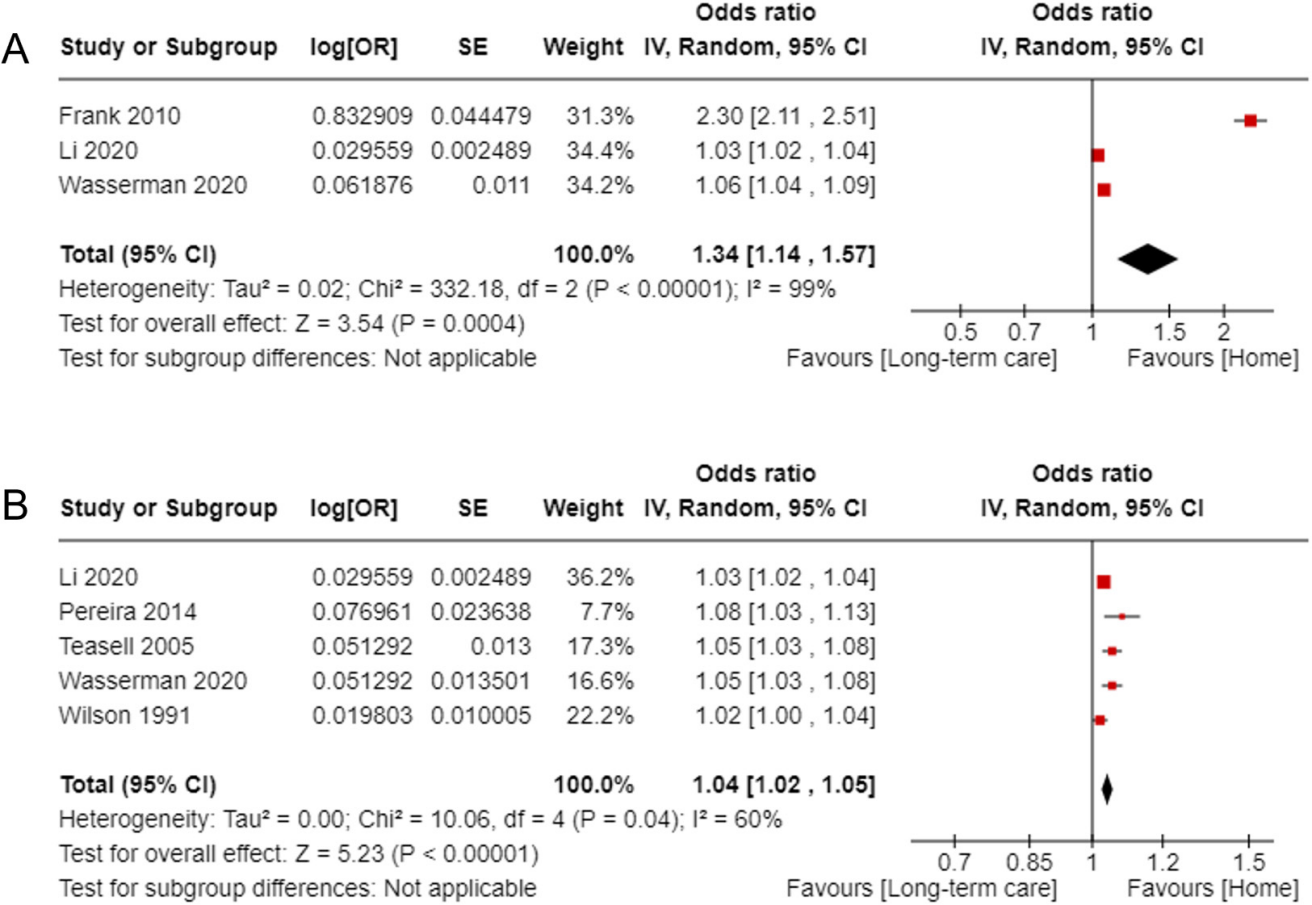
Forest plot for the total Functional Independence Measure on admission to predict returning home: (a) pooled results of studies reporting odds ratio and (b) pooled results of studies reporting adjusted odds ratio.

*Activities of daily living including motor and cognitive domains on admission:* 22 studies^20–24,26–28,31,32,38,42,44–48,50,53–55,60^ assessed the total Functional Independence Measure on admission. A meta-analysis of odds ratio from three studies^20,27,32^ of moderate and high quality (score 9 n = 1, score 8 n = 1, score 7 n = 1) (Figure 3(a)) showed significant results with high heterogeneity (odds ratio 1.34, 95% confidence interval: 1.14–1.57, *I*^2^ = 99%). The pooled result was also significant when the outlier^27^ was removed (odds ratio = 1.04, 95% confidence interval: 1.01–1.08, *I*^2^ =^ ^91%). Five studies^20,32,45,55,62^ of moderate and high quality (score 9 n = 2, score 8 n = 1, score 7 n = 2) with adjusted odds ratio showed significant pooled results (odds ratio = 1.04, 95% confidence interval: 1.02–1.05, *I*^2^ = 60%) for a home discharge (Figure 3(b)). Three threshold values 47,^44^ 60,^47^ and 71^23^ showed consistent results wherein all patient groups above the threshold values had a higher likelihood of home discharge. Subgroup comparison showed that the patients with low scores on admission were less likely to return home compared to the patients with intermediate and high scores.^24,42,53^ Item scores were reported in only three studies^21,44,60^ showing insufficient and inconsistent results. Lastly, one study reported the effect of patients’ daily activity levels measured with the Frenchay Activities Index on discharge destination,^52^ which was not significant (odds ratio =1.02, 95% confidence interval: 0.90–1.16).

*Motor activity on admission:* Revised version of the Ability for Basic Movement Scale,^56^ Motor Assessment Scale,^9,51^ and the Trunk Control Test^28,30,37^ showed significant results for discharge home. Seven studies^8,17,18,20,21,27,38,48^ used balance assessment to predict discharge destination and showed inconsistent results for the Berg Balance Scale total score. When categories were used,^48^ patients with high or intermediate scores had a higher probability of home discharge. For patients without support at home, the admission Berg Balance Scale score was significant in one study.^17^ The measurability of the Berg Balance Scale on admission was a significant predictor of discharge destination.^20^ Sitting and standing balance independence were significant predictive factors of home discharge.^27^ However, only sitting balance was significant in a multivariate analysis. Contradictory results of walking ability on admission were reported for the return home; with nonsignificant results shown for ambulatory status^21^ and significant results shown for the ability to walk (≥ 2 on Motor Assessment Scale gait item)^9^ and walking independently 10 m (with or without a walking aid).^27^ The ability to roll^9^ and to transfer^48^ on admission were also predictive of discharge home.

*Activity and body function on admission:* Significant results were found for Simplified Stroke Rehabilitation Assessment of Movement^44^ and Chedoke McMaster Stroke Assessment^21,42^ postural stability scores. Nonsignificant results were found for Chedoke McMaster Stroke Assessment arm, hand, leg, and foot subscores.^21,42^

Body function on admission: Significant results were found for Motricity Index^37^ and Fugl-Meyer Assessment (total and postural stability),^28,35^ but nonsignificant results were found for Stroke Impairment Assessment Set-motor function.^57^ Grip strength was nonsignificant in one study^57^ and significant for men and women subgroups in another study.^58^

*Motor activities of daily living on discharge:* Three studies^31,36,38^ showed positive results of the motor Functional Independence Measure with discharge home. The threshold values (≥ 31)^59^ showed a significant probability of discharge home for patients with scores about it.

*Activities of daily living including motor and cognitive domains on discharge:* The total Functional Independence Measure was assessed in six studies,^29,36,38,40,48,55^ and showed an increased probability of discharge home with higher scores. One study measured a threshold value of 80 on discharge to predict the destination.^23^ Additionally, one study^43^ used the Lucerne ICF-based Multidisciplinary Observation Scale with two different threshold values defined for patients living alone (≥ 158) and for patients living with family (≥ 130).

## Discussion

The meta-analyses showed that motor and total Functional Independence Measure on admission were significant predictive factors for a return home after inpatient stroke rehabilitation. In addition, the systematic review identified independence in motor activities of daily living (independence in motor and cognitive activities of daily living, independence in sitting and standing balance, ability to walk, roll and transfer, and postural stability) as indicators of discharge home.

The meta-analyses showed an increased likelihood of discharge home of 23% and 34% on the motor and total Functional Independence Measure on admission for a one-point increase on the scale. These results align with previous systematic review findings on patients in the United States of America with acute and subacute stroke^6^ and on patients in acute stroke care.^5^ Our meta-analyses add to these prior findings by showing how motor activities of daily living, assessed with motor Functional Independence Measure, are indicative of a patient's home discharge following inpatient stroke rehabilitation.

The current results after inpatient rehabilitation are similar to those after acute care,^4,5^ where functional status was also an indicator for home discharge. In this systematic review, despite the variability of the length of stay in acute and rehabilitation care, which is primarily related to the national healthcare systems, the studies showed a similar influence of the activities of daily living scales on discharge home. Higher independence was a predictive factor for a home discharge regardless of the healthcare system or recovery phase.

The systematic review identified specific motor activities on admission that are associated with a discharge home after inpatient rehabilitation (independent sitting or standing balance,^27^ sufficient balance to measure the Berg Balance Scale,^20^ walking independently 10 m^27^ or walking with or without assistance (scoring 2 or above on the Gait item of the Motor Assessment Scale),^9^ transferring,^48^ or rolling independently (scoring 6 on the Rolling item of the Motor Assessment Scale)^9^). These activities are interesting as they require less time to assess in clinical practice. However, it is essential to consider them with caution because they were assessed in individual studies. We suggest using these activities as a primary indicator of discharge destination before using further indicators such as the motor Functional Independence Measure.

Next to physical functioning, discharge planning requires to assess further variables, such as cognitive functions and socio-environmental factors. The total Functional Independence Measure assesses cognitive functions in addition to motor functions related to independence in activities of daily living. Cognitive functions are also modifiable factors that have been shown to individually predict discharge destinations in patients with stroke.^63,64^ Patients with better cognitive function are more likely to return home after acute^4,63^ and subacute^57^ care compared to patients with lower cognitive functions. Another significant variable is the prestroke living situation (living alone or together). Patients living alone must demonstrate higher independence in the activities of daily living to return home after a stroke. This concern was addressed in the included study assessing the Lucerne ICF-based Multidisciplinary Observation Scale, where different threshold values were defined for patients living alone (≥ 158) and those living with family (≥ 130). In our previous systematic review and meta-analysis on socio-environmental factors predicting home discharge,^7^ the support of family members was identified as one of the most significant predictive factors for home discharge.

In included studies, threshold values were defined on admission for motor^32,44,49^ (≥ 26,  ≥ 27, and ≥ 39), and total Functional Independence Measure^23,24,42,44,47,53^ (≥ 40,  ≥ 47,  ≥ 60 ≥ 71), and for Berg Balance Scale^8,48^ (≥ 20), and on discharge for motor^59^ (≥ 31) and total Functional Independence Measure^23,24^ (≥ 80), showing that patients with high scores were more likely to be discharged home than patients with low scores. In comparison to the predictive value of continuous scale, the likelihood of home discharge was higher with threshold values. This is clinically relevant as a threshold may be easier to interpret than continuous values in daily clinical practice. A threshold of 29 on the Berg Balance Scale was used to predict community walking speed after stroke.^65^ To define the most appropriate threshold values for discharge destination, an individual patient data meta-analysis could be performed if research datasets are available. Based on the systematic review, we suggest the following threshold values on admission: 20 on Berg Balance Scale, and 26 on the motor Functional Independence Measure.

In this systematic review, the outcome and population were defined broadly to include any potential factors and to be representative of stroke rehabilitation. The eligibility criteria can explain heterogeneity in the meta-analyses. In addition, the variation in healthcare systems influenced the onset to admission interval and length of stay in included studies. Further, sources of methodological heterogeneity were present in the studies, including study design (retrospective vs. prospective), methods of analysis, and adjustments for confounders.^15^

Some limitations of this systematic review must be discussed. Eight of the 45 included studies were identified through reference list manual searching. Although the search strategy was carefully developed, some keywords were missing and should be considered in future research: “home discharge,” and “return home” for discharge planning, and “functional recovery,” “recovery of function,” “Functional Independence Measure” for physical functioning. We observed various quality of data reporting in included studies that limited the number of studies for meta-analysis. This concern is known as a limit of prognostic studies.^66^ However, the quality of prognostic studies has been improving since the introduction of REMARK^67^ guidelines.^15^ A further limitation is the higher representation of studies conducted in the United States of America compared to other countries (14/42 included studies). These studies typically assessed Functional Independence Measure; only one included further physical function outcome measures. Nevertheless, these studies were not overrepresented in meta-analyses.

To summarize, the systematic review showed that higher independence in motor activities of daily living, walking, sitting and standing balance, rolling, transferring, and combined motor and cognitive activities of daily living were strongly associated with discharge home in poststroke patients following inpatient rehabilitation. The meta-analyses showed a significantly increased likelihood of home discharge with higher motor and total Functional Independence Measure scores, with a 24% increase in the likelihood of discharge home with one additional point on the motor Functional Independence Measure scale. Health professionals can assess the ability of their patients to roll, sit, stand, transfer, and walk as a first and quick evaluation to inform discharge planning. Assessments of the motor and total Functional Independence Measure will provide further indication along with other significant factors, such as living together and cognitive functions. Although more research is needed to determine the most appropriate threshold values, clinicians can use on admission a threshold of 20 on the Berg Balance Scale, and of 26 on the motor Functional Independence Measure and on discharge, a threshold of 31 and 80 on the motor and total Functional Independence Measure to differentiate between patient with good potential to return to independence living, and those for whom community living is expected to pose greater risks and challenges.

**Clinical messages**

- Motor and total Functional Independence Measure are associated with discharge destination, as well as cognitive and socio-environmental factors.
- Admission scores above 20 on Berg Balance Scale or 26 on the motor Functional Independence Measure indicate a likelihood of returning home.
- Additional indicators include independence in rolling, sitting, standing, transferring, or walking on admission.

## Supplemental Material

sj-pdf-1-cre-10.1177_02692155231185446 - Supplemental material for Physical functioning factors predicting a return home after stroke rehabilitation: A systematic review and meta-analysis Click here for additional data file. Supplemental material, sj-pdf-1-cre-10.1177_02692155231185446 for Physical functioning factors predicting a return home after stroke rehabilitation: A systematic review and meta-analysis by Odile Chevalley, Steven Truijen, Emmanuelle Opsommer and Wim Saeys in Clinical Rehabilitation

sj-pdf-2-cre-10.1177_02692155231185446 - Supplemental material for Physical functioning factors predicting a return home after stroke rehabilitation: A systematic review and meta-analysis Click here for additional data file. Supplemental material, sj-pdf-2-cre-10.1177_02692155231185446 for Physical functioning factors predicting a return home after stroke rehabilitation: A systematic review and meta-analysis by Odile Chevalley, Steven Truijen, Emmanuelle Opsommer and Wim Saeys in Clinical Rehabilitation

sj-pdf-3-cre-10.1177_02692155231185446 - Supplemental material for Physical functioning factors predicting a return home after stroke rehabilitation: A systematic review and meta-analysis Click here for additional data file. Supplemental material, sj-pdf-3-cre-10.1177_02692155231185446 for Physical functioning factors predicting a return home after stroke rehabilitation: A systematic review and meta-analysis by Odile Chevalley, Steven Truijen, Emmanuelle Opsommer and Wim Saeys in Clinical Rehabilitation

sj-pdf-4-cre-10.1177_02692155231185446 - Supplemental material for Physical functioning factors predicting a return home after stroke rehabilitation: A systematic review and meta-analysis Click here for additional data file. Supplemental material, sj-pdf-4-cre-10.1177_02692155231185446 for Physical functioning factors predicting a return home after stroke rehabilitation: A systematic review and meta-analysis by Odile Chevalley, Steven Truijen, Emmanuelle Opsommer and Wim Saeys in Clinical Rehabilitation
