# Supplementary material for: Physical functioning factors predicting a return home after stroke rehabilitation: A systematic review and meta-analysis
Source: Clin Rehabil. 2023 Jul 10;37(12):1698–716. doi: 10.1177/02692155231185446 (PMC10580673; doi:10.1177/02692155231185446)
Supplement: sj-pdf-2-cre-10.1177_02692155231185446 - Supplemental material for Physical functioning factors predicting a return home after stroke rehabilitation: A systematic review and meta-analysis [file sj-pdf-2-cre-10.1177_02692155231185446.pdf]

Search strategy for each database

In the databases with Boolean searches (PubMed, Embase, CINAHL, Cochrane Library, and Web of Science), each group of keywords was searched individually in the respective databases and then combined in the advance search option with the Boolean “AND”.

PubMed

(“stroke”[Mesh] OR "Stroke Rehabilitation"[MeSH] OR “cerebrovascular lesion*”[tiab] OR “apoplex*”[tiab] OR “brain attack*”[tiab] OR “brain infarction*”[tiab] OR “brain insult*”[tiab] OR “brain ischemic attack*”[tiab] OR ” brain ischemia”[tiab] OR “brain ischaemia”[tiab] OR “brain vascular accident*”[tiab] OR “cerebral insult*”[tiab] OR “cerebral vascular accident*”[tiab] OR “cerebrovascular accident*”[tiab] OR “cerebrovascular disorder*”[tiab] OR “cerebrovascular insult*”[tiab] OR “cva”[tiab] OR “intracranial haemorrhage”[tiab] OR “intracranial hemorrhage”[tiab] OR “ischaemic cerebral attack*”[tiab] OR “ischemic cerebral attack*”[tiab] OR “ischemic seizure*”[tiab] OR “stroke*”[tiab] OR “stroke rehabilitation*”[tiab] OR “hypoxia-ischemia”[tiab] OR “cerebral haemorrhage”[tiab] OR “cerebral hemorrhage”[tiab] OR “cerebral infarction”[tiab] OR “brain haemorrhage”[tiab] OR “brain hemorrhage”[tiab])

("Patient Discharge"[Mesh] OR "Patient Transfer"[Mesh] OR “discharge course*”[tiab] OR “discharge decision*”[tiab] OR “discharge destination*”[tiab] OR “discharge disposition*”[tiab] OR “discharge location*”[tiab] OR “discharge planning*”[tiab] OR “hospital discharge*”[tiab] OR “patient discharge*”[tiab] OR “patient transfer*”[tiab])

("Activities of Daily Living"[Mesh] OR "Walking"[Mesh] OR "Somatosensory Disorders"[Mesh] OR "Movement"[Mesh] OR “pain”[Mesh] OR “proprioception”[Mesh] OR “activities of daily living”[tiab] OR “ADL”[tiab] OR “ambulation”[tiab] OR “daily life activit*”[tiab] OR “Daily Living Activit*”[tiab] OR “Functional status”[tiab] OR “gait”[tiab] OR “hypaesthesia”[tiab] OR “hyperaesthesia”[tiab] OR “Hyperalgesia”[tiab] OR “Hyperesthesia”[tiab] OR “Hypesthesia”[tiab] OR “Independent Living”[tiab] OR “light touch”[tiab] OR “locomotion”[tiab] OR “movement*”[tiab] OR “pain”[tiab] OR “paraesthesia”[tiab] OR “Paresthesia”[tiab] OR “Pinprick”[tiab] OR “postural balance”[tiab] OR “pressure”[tiab] OR “proprioception”[tiab] OR “Proprioceptive Disorder*”[tiab] OR “Self Care*”[tiab] OR “Self-Neglect*”[tiab] OR “sensory dysfunction”[tiab] OR “Social Participation”[tiab] OR “Somatosensory Disorder*”[tiab] OR “stair climbing”[tiab] OR “temperature”[tiab] OR “thermal sens*”[tiab] OR “walking”[tiab])

EMBASE

(“cerebrovascular accident”/exp OR “stroke patient”/exp OR “stroke rehabilitation”/exp OR (“cerebrovascular lesion*” OR “apoplex*” OR “brain attack*” OR “brain infarction*” OR “brain insult*” OR “brain ischaemic attack*” OR “brain ischemic attack*” OR “brain ischaemia” OR “brain ischemia” OR “brain vascular accident*” OR “cerebral insult*” OR “cerebr* vascular accident*” OR “cerebrovascular accident*” OR “cerebrovascular disorder*” OR “cerebrovascular insult*” OR “cva” OR “intracranial haemorrhage” OR “intracranial hemorrhage” OR “ischaemic cerebral attack*” OR “ischaemic seizure*” OR “ischemic cerebral attack*” OR “ischemic seizure*” OR “stroke*” OR “stroke rehabilitation*” OR “hypoxia-ischemia” OR “cerebral haemorrhage” OR “cerebral hemorrhage” OR “cerebral infarction” OR “brain haemorrhage” OR “brain hemorrhage”):ti,ab,kw)

("hospital discharge"/exp OR (“discharge course*” OR “discharge decision*” OR “discharge destination*” OR “discharge disposition*” OR “discharge location*” OR “discharge planning*” OR “hospital discharge*” OR “patient discharge*” OR “patient transfer*”):ti,ab,kw)

("daily life activity"/exp OR “independent living”/exp OR “self care”/exp OR “functional status”/exp OR “self neglect”/exp OR “social participation”/exp OR "walking"/exp OR "somatosensory disorder"/exp OR "movement (physiology)"/exp OR “proprioception”/exp OR “pain”/exp OR ("activities of daily living” OR "ADL” OR "ambulation” OR "daily life activit*” OR "Daily Living Activit*” OR "Functional status” OR "gait” OR "hypaesthesia” OR "hyperaesthesia” OR "Hyperalgesia” OR "Hyperesthesia” OR "Hypesthesia” OR "Independent Living” OR "light touch” OR "locomotion” OR "movement*” OR “pain” OR "paraesthesia” OR "Paresthesia” OR "Pinprick” OR “postural balance” OR “pressure” OR "proprioception” OR "Proprioceptive Disorder*” OR "Self Care*” OR "Self-Neglect” OR "sensory dysfunction” OR "Social Participation” OR "Somatosensory Disorder*” OR "stair climbing” OR "temperature” OR "thermal sens*” OR "walking”):ti,ab,kw)

CINAHL

(MH "Stroke+" OR MH "Stroke Patients" OR TI(“cerebrovascular lesion*” OR “apoplex*” OR “brain attack*” OR “brain infarction*” OR “brain insult*” OR “brain ischaemic attack*” OR “brain ischemic attack*” OR “brain ischaemia” OR “brain ischemia” OR “brain vascular accident*” OR “cerebral insult*” OR “cerebr* vascular accident*” OR “cerebrovascular accident*” OR “cerebrovascular disorder*” OR “cerebrovascular insult*” OR “CVA” OR “intracranial haemorrhage” OR “intracranial hemorrhage” OR “ischaemic cerebral attack*” OR “ischaemic seizure*” OR “ischemic cerebral attack*” OR “ischemic seizure*” OR “stroke*” OR “stroke rehabilitation*” OR “hypoxia-ischemia” OR “cerebral haemorrhage” OR “cerebral hemorrhage” OR “cerebral infarction” OR “brain haemorrhage” OR “brain hemorrhage”) OR AB(“cerebrovascular lesion*” OR “apoplex*” OR “brain attack*” OR “brain infarction*” OR “brain insult*” OR “brain ischaemic attack*” OR “brain ischemic attack*” OR “brain ischaemia” OR “brain ischemia” OR “brain vascular accident*” OR “cerebral insult*” OR “cerebr* vascular accident*” OR “cerebrovascular accident*” OR “cerebrovascular disorder*” OR “cerebrovascular insult*” OR “CVA” OR “intracranial haemorrhage” OR “intracranial hemorrhage” OR “ischaemic cerebral attack*” OR “ischaemic seizure*” OR “ischemic cerebral attack*” OR “ischemic seizure*” OR “stroke*” OR “stroke rehabilitation*” OR “hypoxia-ischemia” OR “cerebral haemorrhage” OR “cerebral hemorrhage” OR “cerebral infarction” OR “brain haemorrhage” OR “brain hemorrhage”))

(MH "Patient Discharge+" OR TI (“discharge course*” OR “discharge decision*” OR “discharge destination*” OR “discharge disposition*” OR “discharge location*” OR “discharge planning*” OR “hospital discharge*” OR “patient discharge*” OR “patient transfer*”) OR AB (“discharge course*” OR “discharge decision*” OR “discharge destination*” OR “discharge disposition*” OR “discharge location*” OR “discharge planning*” OR “hospital discharge*” OR “patient discharge*” OR “patient transfer*”))

(MH "Activities of Daily Living+" OR MH "Movement+" OR MH "Walking+" OR MH "Somatosensory Disorders+" OR MH “pain” OR MH “proprioception” OR TI ("activities of daily living” OR "ADL” OR "ambulation” OR "daily life activit*” OR "Daily Living Activit*” OR "Functional status” OR "gait” OR "hypaesthesia” OR "hyperaesthesia” OR "Hyperalgesia” OR "Hyperesthesia” OR "Hypesthesia” OR "Independent Living” OR "light touch” OR "locomotion” OR "movement*” OR "pain” OR "paraesthesia” OR "Paresthesia” OR "Pinprick” OR “postural balance” OR "pressure” OR "proprioception” OR "Proprioceptive Disorder*” OR "Self Care*” OR "Self-Neglect” OR "sensory dysfunction” OR "Social Participation” OR "Somatosensory Disorder*” OR "stair climbing” OR "temperature” OR "thermal sens*” OR "walking”) OR AB ("activities of daily living” OR "ADL” OR "ambulation” OR "daily life activit*” OR "Daily Living Activit*” OR "Functional status” OR "gait” OR "hypaesthesia” OR "hyperaesthesia” OR "Hyperalgesia” OR "Hyperesthesia” OR "Hypesthesia” OR "Independent Living” OR "light touch” OR "locomotion” OR "movement*” OR "pain” OR "paraesthesia” OR "Paresthesia” OR "Pinprick” OR “postural balance” OR "pressure” OR "proprioception” OR "Proprioceptive Disorder*” OR "Self Care*” OR "Self-Neglect” OR "sensory dysfunction” OR "Social Participation” OR "Somatosensory Disorder*” OR "stair climbing” OR "temperature” OR "thermal sens*” OR "walking”))

Cochrane Library

(“cerebrovascular lesion*” OR “apoplex*” OR “brain attack*” OR “brain infarction*” OR “brain insult*” OR “brain ischaemic attack*” OR “brain ischemic attack*” OR “brain ischaemia” OR “brain ischemia” OR “brain vascular accident*” OR “cerebral insult*” OR “cerebr* vascular accident*” OR “cerebrovascular accident*” OR “cerebrovascular disorder*” OR “cerebrovascular insult*” OR “CVA” OR “intracranial haemorrhage” OR “intracranial hemorrhage” OR “ischaemic cerebral attack*” OR “ischaemic seizure*” OR “ischemic cerebral attack*” OR “ischemic seizure*” OR “stroke*” OR “stroke rehabilitation*” OR “hypoxia-ischemia” OR “cerebral haemorrhage” OR “cerebral hemorrhage” OR “cerebral infarction” OR “brain haemorrhage” OR “brain hemorrhage”):ti,ab,kw

(“discharge course*” OR “discharge decision*” OR “discharge destination*” OR “discharge disposition*” OR “discharge location*” OR “discharge planning*” OR “hospital discharge*” OR “patient discharge*” OR “patient transfer*”):ti,ab,kw

("activities of daily living” OR "ADL” OR "ambulation” OR "daily life activit*” OR "Daily Living Activit*” OR "Functional status” OR "gait” OR "hypaesthesia” OR "hyperaesthesia” OR "Hyperalgesia” OR "Hyperesthesia” OR "Hypesthesia” OR "Independent Living” OR "light touch” OR "locomotion” OR "movement*” OR "pain” OR "paraesthesia” OR "Paresthesia” OR "Pinprick” OR “postural balance” OR "pressure” OR "proprioception” OR "Proprioceptive Disorder*” OR "Self Care*” OR "Self-Neglect” OR "sensory dysfunction” OR "Social Participation” OR "Somatosensory Disorder*” OR "stair climbing” OR "temperature” OR "thermal sens*” OR "walking”):ti,ab,kw

Web of Science

TS=(“cerebrovascular lesion*” OR “apoplex*” OR “brain attack*” OR “brain infarction*” OR “brain insult*” OR “brain ischaemic attack*” OR “brain ischemic attack*” OR “brain ischaemia” OR “brain ischemia” OR “brain vascular accident*” OR “cerebral insult*” OR “cerebr* vascular accident*” OR “cerebrovascular accident*” OR “cerebrovascular disorder*” OR “cerebrovascular insult*” OR “CVA” OR “intracranial haemorrhage” OR “intracranial hemorrhage” OR “ischaemic cerebral attack*” OR “ischaemic seizure*” OR “ischemic cerebral attack*” OR “ischemic seizure*” OR “stroke*” OR “stroke rehabilitation*” OR “hypoxia-ischemia” OR “cerebral haemorrhage” OR “cerebral hemorrhage” OR “cerebral infarction” OR “brain haemorrhage” OR “brain hemorrhage”)

TS=(“discharge course*” OR “discharge decision*” OR “discharge destination*” OR “discharge disposition*” OR “discharge location*” OR “discharge planning*” OR “hospital discharge*” OR “patient discharge*” OR “patient transfer*”)

TS=("activities of daily living” OR "ADL” OR "ambulation” OR "daily life activit*” OR "Daily Living Activit*” OR "Functional status” OR "gait” OR "hypaesthesia” OR "hyperaesthesia” OR "Hyperalgesia” OR "Hyperesthesia” OR "Hypesthesia” OR "Independent Living” OR "light touch” OR "locomotion” OR "movement*” OR "pain” OR "paraesthesia” OR "Paresthesia” OR "Pinprick” OR “postural balance” OR "pressure” OR "proprioception” OR "Proprioceptive Disorder*” OR "Self Care*” OR "Self-Neglect” OR "sensory dysfunction” OR "Social Participation” OR "Somatosensory Disorder*” OR "stair climbing” OR "temperature” OR "thermal sens*” OR "walking”)

PEDro

The following keywords were introduced into the search field “Abstract and Title”. The results of the nine searches were combined and duplicates were removed.

(1) Stroke AND patient discharge
(2) Stroke AND discharge planning
(3) Stroke AND discharge decision
(4) Stroke AND discharge destination
(5) Stroke AND discharge location
(6) Stroke AND discharge course
(7) Stroke AND discharge disposition
(8) Stroke AND hospital discharge
(9) Stroke AND patient transfer
