# Supplementary material for: Physical functioning factors predicting a return home after stroke rehabilitation: A systematic review and meta-analysis
Source: Clin Rehabil. 2023 Jul 10;37(12):1698–716. doi: 10.1177/02692155231185446 (PMC10580673; doi:10.1177/02692155231185446)
Supplement: sj-pdf-3-cre-10.1177_02692155231185446 - Supplemental material for Physical functioning factors predicting a return home after stroke rehabilitation: A systematic review and meta-analysis [file sj-pdf-3-cre-10.1177_02692155231185446.pdf]

Supplementary File 3

Methodological assessment with NOS for case-control and cohort studies

Study ID	Newcastle Ottawa Scale items – case-control studies								
	1.1	1.2	1.3	1.4	2	3.1	3.2	3.3	Total
Davidoff 1992 (ISR)	*	*	*	*	*		*	*	7
Wasserman 2020 (CAN)	*	*	*	*		*	*	*	7

1.1: Is the case definition adequate; 1.2: Representativeness of the cases; 1.3: Selection of Controls; 1.4: Definition of Controls; 2: Comparability of cases and controls on the basis of the design or analysis; 3.1: Ascertainment of exposure; 3.2: Same method of ascertainment for cases and controls; 3.3: Non-Response rate

Study ID	Newcastle Ottawa Scale items – cohort studies								
	1.1	1.2	1.3	1.4	2	3.1	3.2	3.3	Total
Agarwal 2003 (CAN)	*	*	*	*	**	*	*	*	9
Alexander 1994 (USA)	*	*	*	*			*	*	6
Black 1999 (USA)	*	*	*	*		*	*	*	7
Bottemiller 2006 (USA)	*	*	*	*		*	*	*	7
Brauer 2008 (AUS)	*	*	*	*	**	*	*	*	9
Brown 2015 (USA)	*	*	*	*	**	*	*	*	9
Denti 2008 (ITA)	*	*	*	*	**	*	*	*	9
Frank 2010 (CHE)	*	*	*	*	**	*	*	*	9
Gialanella 2012 (ITA)	*	*	*	*	*		*	*	7
Granger 1992 (USA)	*	*	*	*		*	*	*	7
Hirano 2017 (JPN)	*	*	*	*	*		*	*	7
Ito 2022 (JPN)	*	*	*	*	**		*	*	8
Koyama 2011 (JPN)	*	*	*	*		*	*	*	7
Li 2020 (Hong-Kong)		*	*	*	**	*	*	*	8
Ling 2004 (CHN)	*	*	*	*	**	*	*	*	9
Löfgren 1997 (SWE)	*	*	*	*		*	*	*	7
Löfgren 2000 (SWE)	*	*	*	*		*	*	*	7
Maeshima 2016 (JPN)	*	*	*	*		*	*	*	7
Massucci 2006 (ITA)	*	*	*	*	*	*	*	*	8
Matsushita 2022 (JPN)		*	*	*	**	*	*	*	8
Miura 2018 (JPN)	*	*	*	*	**	*	*	*	9
Mokler 2000 (USA)	*	*		*			*		4
Mutai 2012 (JPN)	*	*	*	*	**	*	*	*	9
Ng 2005 (USA)		*	*	*	**	*	*	*	8
Nguyen 2015 (USA)	*	*	*	*	**	*	*	*	9
Oczkowski 1993 (CAN)	*	*	*	*	**		*		7
Onishi 2022 (JPN)		*	*	*	**	*	*	*	8
Ottiger 2020 (CHE)	*	*	*	*	**	*	*	*	9
Ouellette 2015 (USA)	*	*	*	*		*	*		6
Pereira 2014 (USA)		*	*	*	**	*	*	*	8
Petrilli 2002 (FRA)		*	*	*		*	*	*	6
Pohl 2013 (USA)	*	*	*	*		*	*	*	7
Saab 2019 (CAN)	*	*	*	*	**	*	*	*	9
Tanwir 2014 (CAN)	*	*	*	*		*	*	*	7
Teasell 2005 (CAN)	*	*	*	*	**	*	*	*	9
Tucak 2010 (AUS)		*	*	*	**	*	*	*	8
Vluggen 2020 (NLD)		*	*	*		*	*	*	6
Wee 1999 (CAN)	*	*	*	*	*	*	*	*	8
Wee 2003 (CAN)	*	*	*	*	**	*	*	*	9
Wee 2005 (CAN)	*	*	*	*	*	*	*	*	8
Ween 1996 (USA)	*	*	*	*		*	*	*	7
Ween 2000 (USA)	*	*	*	*		*	*	*	7
Wilson 1991 (USA)	*	*	*	*		*	*	*	7
Yang 2020 (JPN)	*	*	*	*			*	*	6

1.1: Representativeness of the exposed cohort; 1.2: Selection of the non exposed cohort; 1.3: Ascertainment of exposure; 1.4: Demonstration that outcome of interest was not present at start of study; 2: Comparability of cohorts on the basis of the design or analysis; 3.1: Assessment of outcome; 3.2: Was follow-up long enough for outcomes to occur; 3.3: Adequacy of follow up of cohorts
