# Supplementary material for: Physical functioning factors predicting a return home after stroke rehabilitation: A systematic review and meta-analysis
Source: Clin Rehabil. 2023 Jul 10;37(12):1698–716. doi: 10.1177/02692155231185446 (PMC10580673; doi:10.1177/02692155231185446)
Supplement: sj-pdf-4-cre-10.1177_02692155231185446 - Supplemental material for Physical functioning factors predicting a return home after stroke rehabilitation: A systematic review and meta-analysis [file sj-pdf-4-cre-10.1177_02692155231185446.pdf]

Supplementary material 4

Tables of results of studies that were not included in meta-analyses.

Table 1a: Motor ADL scales on admission

Outcome measure (Scale, subscale)	Study ID	*n* discharge		Odds ratio		Adjusted odds ratio		Narrative
		Home	Other	Odds Ratio	95% Confidence Interval	Adjusted Odds Ratio	95% Confidence Interval	
Motor Functional Independence Measure	Brown 2015	103857	44510		Significant		Significant	
	Denti 2008	287	72				Non-significant	
	Gialanella 2013	205	36	1.25	Significant		Non-significant	
	Maeshima 2016	71	18					Significant group difference
	Mutai 2012	151	23				Non-significant	
	Wasserman 2020	180	60		Significant		Significant	Similar to total score on admission
Motor Functional Independence Measure, cut-off 26	Ouellette 2015	297	110	4.20	3.64-4.85			
Motor Functional Independence Measure, intermediate vs. low (39-50 vs <39)	Tanwir 2014			2.48	1.04-5.91			
Motor Functional Independence Measure, intermediate vs. low (27-52 vs <27)	Li 2020	304	258	3.04	2.01-4.60			
Motor Functional Independence Measure, high vs. low (>50 vs <39)	Tanwir 2014			4.75	2.14-10.54			
Motor Functional Independence Measure, high vs. low (>52 vs <27)	Li 2020	304	258	5.10	3.25-8.00			
Motor Functional Independence Measure, Self-care	Frank 2010	828	469	2.10	1.90-2.32		Non-significant	
	Ling 2004	789	322			1.06	1.01-1.11	
Motor Functional Independence Measure, Locomotion	Frank 2010	828	469	1.60	1.50-1.71		Non-significant	
	Ling 2004	789	322			1.21	1.06-1.39	
Motor Functional Independence Measure, Transfer	Frank 2010	828	469	1.80	1.70-1.91		Non-significant	
Motor Functional Independence Measure, Bed/chair transfer	Mokler 2000	115	144				Significant	
Motor Functional Independence Measure, Upper body dressing	Mokler 2000	115	144				Significant	

Katz-15	Löfgren 1997	53	47					Significant group difference
	Löfgren 2000	71	31					Significant group difference
	Vluggen 2020	71	21	0.70	0.53-0.92			
Kenny self-care	Davidoff 1992	141	51			1.10	0.78-1.57	
Barthel Index, mobility	Löfgren 1997	53	47					Significant group difference

n: number of participants.

Table 1b: Motor ADL scales on discharge

**Outcome measure**	**Study ID**	***n* discharge**		**Odds ratio**		**Adjusted odds ratio**		**Narrative**
**(Scale, subscale)**		**Home**	**Other**	**Odds Ratio**	**95% Confidence Interval**	**Adjusted Odds Ratio**	**95% Confidence Interval**	
Motor Functional Independence Measure	Koyama 2011	123	40					
	Maeshima 2016	71	18					Significant group difference
	Miura 2018	175	167	1.18	1.14-1.23	1.10	1.04-1.18	
Motor Functional Independence Measure, cut-off 31	Onishi 2022	147	58					Group difference

n: number of participants.

Table 2a: Motor and cognitive ADL scales on admission

**Outcome measure**	**Study ID**	***n* discharge**		**Odds ratio**		**Adjusted Odds ratio**		**Narrative**
**(Scale, subscale)**		**Home**	**Other**	**Odds Ratio**	**95% Confidence Interval**	**Adjusted Odds Ratio**	**95% Confidence Interval**	
Total Functional Independence Measure	Agarwal 2003	80	24					Significant group difference
	Alexander 1994	361	103					Significant factor
	Gialanella 2012	205	36	1.32	Non-significant			
	Denti 2008	287	72				Non-significant	
	Maeshima 2016	71	18					Significant group difference
	Petrilli 2002	81	11					Significant group difference
	Saab 2019			2.36	Significant			
	Ween 2000	167	77					Significant factor
Total Functional Independence Measure, cut-off 47	Ouellette 2015	297	110	3.90	2.40-6.34			
Total Functional Independence Measure, cut-off 60	Pohl 2013	24035	7884			5.84	5.51-6.20	
Total Functional Independence Measure, cut-off 71	Black 1999	168	66					Significant group difference
Total Functional Independence Measure, subgroups (low, intermediate, high)	Oczkowski 1993	80	30					Higher likelihood with higher score
Total Functional Independence Measure, subgroups (<40, 40-59, 60-80, >80)	Ween 1996	223	81					Significant group difference
Total Functional Independence Measure, subgroups (<40, 40-80, >80)	Bottemiller 2006	489	259					Group difference

n: number of participants.

Supplementary material 4

Table 2b: Motor and cognitive ADL scales on discharge

Outcome measure	Study ID	*n* discharge		Odds ratio		Adjusted Odds ratio		Narrative
(Scale, subscale)		Home	Other	Odds Ratio	95% Confidence Interval	Adjusted Odds Ratio	95% Confidence Interval	
Total Functional Independence Measure	Granger 1992	5909	1769					Higher probability with higher scores
	Maeshima 2016	71	18					Significant group difference
	Miura 2018	175	167	1.27	1.21-1.33	1.12	1.08-1.17	
	Ng 2005	55	34	1.04	1.01-1.07			
	Saab 2019			2.69	Significant		Non-significant	
	Wilson 1991	212	70			1.03	Significant	
Lucerne ICF-based Multidisciplinary Observation Scale, cut-off 158 for patient alone	Ottiger 2020	94	43			292.51	52.00-1645.53	
Lucerne ICF-based Multidisciplinary Observation Scale, cut-off 130 for patients with family	Ottiger 2020	374	44			89.40	32.30-247.42	

n: number of participants.

Supplementary material 4

Table 3a: Motor activity scales on admission

Outcome measure (Scale, subscale)	Study ID	*n* discharge		Odds ratio		Adjusted odds ratio		Narrative
		Home	Other	Odds Ratio	95% Confidence Interval	Adjusted Odds Ratio	95% Confidence Interval	
Berg Balance Scale	Agarwal 2003	80	24					Significant difference between group
	Wasserman 2020	180	60					Non-significant correlation with discharge destination
	Wee 2005	246	67	1.09	Significant			
	Wee 1999	98	30			1.09	1.04-1.13	
Berg Balance Scale for patients without support	Wee 2003	246	67			1.09	1.06-1.12	
Berg Balance Scale, measurability	Wasserman 2020	180	60	7.14	3.57-14.29	5.88	2.22-15.57	
Berg Balance Scale, score 20-40 vs. <20	Saab 2019			2.46	Significant			
Berg Balance Scale, score >40 vs <20	Saab 2019			5.85	Significant			
Sitting balance	Frank 2010	828	469	9.1	6.4-12.9	1.8	1.1-2.8	
Standing balance	Frank 2010	828	469	5.7	4.5-7.3			
Independent walking ability 10 m	Frank 2010	828	504	4.4	3.4-5.8			
Revised version of the Ability for Basic Movement Scale	Yang 2020	61	33	1.24	1.13-1.36			
Motor Assessment Scale	Tucak 2010	169	70			1.12	1.08-1.15	
Motor Assessment Scale, cut-off 30	Tucak 2010	169	70	9.13	4.37-19.09			
Motor Assessment Scale, Rolling	Brauer 2008	410	136			1.28	1.11-1.49	
Motor Assessment Scale, Gait	Brauer 2008	410	136			1.67	1.28-2.27	
Motor Assessment Scale, Rolling, cut-off 6	Brauer 2008	410	136			2.63	1.27-5.88	
Motor Assessment Scale, Gait, cut-off 2	Brauer 2008	410	136			6.25	3.13-14.28	
Trunk Control Test	Gialanella 2012	205	36	1.26	Significant			
	Hirano 2017	61	19				Significant	
	Massucci 2006			1.01	1.00-1.02			

n: number of participants.

Supplementary material 4

Table 3b: Motor activity scales on discharge

**Outcome measure**<br><br>**(Scale, subscale)**	**Study ID**	***n* discharge**		**Odds ratio**		**Adjusted odds ratio**		**Narrative**
		**Home**	**Other**	**Odds Ratio**	**95% Confidence Interval**	**Adjusted Odds Ratio**	**95% Confidence Interval**	
Berg Balance Scale	Miura 2018	175	167	1.15	1.12-1.19	0.99	0.94-1.05	

n: number of participants.

Supplementary material 4

Table 4: Motor activity and body function scales on admission

Outcome measure (Scale, subscale)	Study ID	*n* discharge		Odds ratio		Narrative
		Home	Other	Odds Ratio	95% Confidence Interval	
Simplified STroke REhabilitation Assessment of Movement	Ouellette 2015	297	110	3.3		
Chedoke McMaster Stroke Assessment, arm	Agarwal 2003	80	24			Non-significant
	Oczkowski 1993	80	30			Non-significant
Chedoke McMaster Stroke Assessment, hand	Agarwal 2003	80	24			Non-significant
	Oczkowski 1993	80	30			Non-significant
Chedoke McMaster Stroke Assessment, leg	Agarwal 2003	80	24			Non-significant
	Oczkowski 1993	80	30			Non-significant
Chedoke McMaster Stroke Assessment, foot	Agarwal 2003	80	24			Non-significant
	Oczkowski 1993	80	30			Non-significant
Chedoke McMaster Stroke Assessment, postural stability	Agarwal 2003	80	24			Significant group difference
	Oczkowski 1993	80	30			Highly correlated with discharge destination

n: number of participants.

Table 5: Body function scales on admission

Outcome measure (Scale, subscale)	Study ID	*n* discharge		Odds ratio		Adjusted odds ratio		Narrative
		Home	Other	Odds Ratio	95% Confidence Interval	Adjusted Odds Ratio	95% Confidence Interval	
Fugl Meyer Assessment	Gialanella 2012	205	36	1.21	Significant			
Fugl Meyer Assessment, postural stability	Löfgren 1997	53	47			1.69	1.32-2.16	
	Löfgren 2000	71	31				Significant	
Fugl Meyer Assessment, range of motion	Löfgren 1997	53	47					Significant group difference
Fugl Meyer Assessment, motor function	Löfgren 1997	53	47					Significant group difference
Motricity Index, side score	Massucci 2006			1.01	1.00-1.02			
Grip strength	Ito 2022	1011	218			1.03	0.99-1.06	
Grip strength, men	Matsushita 2022	251	117			1.07	1.03-1.10	
Grip strength, women	Matsushita 2022	197	134			1.07	1.00-1.13	
SIAS-m, upper limb	Ito 2022	1011	218			1.03	0.93-1.15	
SIAS-m, lower limb	Ito 2022	1011	218			1.07	0.99-1.16	

n: number of participants.
